# Effect of Microstructure Evolution on the Overall Response of Porous-Plastic Solids

**DOI:** 10.3390/ma3021031

**Published:** 2010-02-04

**Authors:** Stefano Mariani

**Affiliations:** Politecnico di Milano, Dipartimento di Ingegneria Strutturale, Piazza Leonardo da Vinci 32, 20133 - Milano, Italy; E-Mail: stefano.mariani@polimi.it; Tel.: +39-0223994279; Fax: +39-0223994220

**Keywords:** two-phase composites, ductile fracture, porous-plastic materials, voids and inclusions

## Abstract

Ductile fracture is the macroscopic result of a micromechanical process consisting in void nucleation and growth to coalescence. While growing in size, voids also evolve in shape because of the non-uniform deformation field in the surrounding material; this shape evolution is either disregarded or approximately accounted for by constitutive laws for porous-plastic solids. To assess the effect of void distortion on the overall properties of a porous-plastic material prior to any coalescence-dominated event, we here present a micromechanical study in which the void-containing material is treated as a two-phase (matrix and inclusion) composite. A cylindrical representative volume element (RVE), featuring elliptic cross-section and containing a coaxial and confocal elliptic cylindrical cavity, is considered. In case of a matrix obeying $J_{2}$ flow theory of plasticity, the overall yield domain and the evolution laws for the volume fraction and aspect ratio of the void are obtained. Under assigned strain histories, these theoretical findings are then compared to finite element unit-cell simulations, in order to assess the capability of the proposed results to track microstructure evolution. The improvements with respect to the customarily adopted Gurson’s model are also discussed.

## 1. Introduction

Ductile tearing in metals is the macroscopic result of a micromechanical process consisting of void nucleation, and growth to coalescence [1,2]. Elastic-brittle second-phase particles, or inclusions (like, e.g., manganese sulfide in steels [1,3,4,5]), featuring spheroidal or cylindrical shape, trigger the onset of strain localization and therefore lead to diffused damage mechanisms in a formerly fully-dense material. Void nucleation may be caused either by inclusion cracking or debonding of the inclusion from the surrounding metal, and is strongly sensitive to micro-defects; void coalescence instead represents the final stage of the growth process, and consists in the break-down of microligaments between neighboring voids. This latter stage becomes dominant at values of the void volume fraction *f*, which is defined as the ratio between the volume of nucleated voids within a RVE of the material and the volume of the RVE itself [2,6], exceeding a critical threshold usually recognized to amount to f≈0.2.

In this paper we focus on the void growth phase only; readers are referred to [2,7] for details and modeling aspects concerning the nucleation and coalescence stages. The void-containing solid is (virtually) treated as a two-phase composite medium: the matrix material is represented by the fully-dense metal, whereas the inclusions are the voids featuring null elastic and strength properties. Through a rather standard homogenization procedure for nonlinear materials (based on the kinematic approach to limit analysis), the plastic behavior of the porous-plastic solid is obtained in terms of overall yield locus and evolution laws for the state variables describing the shape and size of the voids [8,9,10]. Following a customary approach (see, e.g., [6,11,12,13]), instead of a population of randomly distributed microvoids, each one featuring its own size and shape, a single void is allowed for and assumed to gather the basic properties of the actual microstructure. The effects of void clusters are therefore smeared over the whole RVE.

Microstructure evolution represents a key feature to describe the softening, *i.e.*, the reduction of the overall strength, induced by void growth. Widely adopted models, like the Gurson’s one [2,6], assume that voids always retain their shape while growing; when deviatoric states of stress become dominant, alternative modeling procedures for ductile fracture are applied to achieve accuracy (e.g., see [14]).

Recently, much work has been devoted to the analysis of yielding and microstructure evolution in solids containing spheroidal voids, see e.g., [13,15,16,17,18,19,20]. Gologanu et alii in [13,15] provided a frame to account at the constitutive level for the effects of void shape. Here, we follow a similar procedure for cylindrical microstructures, which turn out to be representative when, e.g., voids nucleate from manganese sulfide inclusions elongated upon rolling. A cylindrical RVE with elliptic cross-section, containing a coaxial and confocal cylindrical void is therefore considered.

A drawback of results available in the literature is represented by the lack of a general setting that allows to furnish results for any degree of anisotropy in void geometry and arrangement. For instance, in [13,15] two different analyses were necessary to allow for prolate and oblate spheroidal voids. In [10] we proposed, and we further discuss here a local coordinate mapping that is able to deal with elliptical voids featuring any orientation of their major axis.

The capabilities of the proposed model, in terms of description of strength degradation due to void growth and distortion at finite strains, are here assessed under plane strain conditions, which occur in the central portion of any fracture process zone (ahead of the tip of a growing crack). We show that microstructure distortion due to deviatoric states of stress can be appropriately accounted for, and the induced softening (not modeled by the Gurson’s model) well described. To check the model accuracy, outcomes of the constitutive law are compared to finite element simulations at the unit-cell level [21,22].

The remainder of this paper is organized as follows. In Section 2 we furnish the fundamentals of the micromechanical procedure to obtain the overall yield locus, and the relevant microstructure evolution for orthotropic porous-plastic materials. In Section 3 the response of void-containing materials at finite strains is investigated. To assess the link between microstructure evolution and overall response, results are compared to those obtained with the Gurson’s model and with finite element unit-cell simulations. Finally, in Section 4 concluding remarks are drawn and possible future enhancements are envisaged.

As far as notation is concerned, a standard component representation for tensors is adopted throughout; summation over repeated indeces will be therefore implicitly assumed. A superposed dot will represent time rates.

## 2. Homogenized Nonlinear Properties of Orthotropic Porous-Ductile Media

Let us consider a two-phase composite, which possesses a statistically uniform microstructure [23,24]. Focusing on a single RVE, we define the macroscopic strain rate ${\dot{E}}_{ij}$ and stress $\Sigma_{ij}$ tensors (where i,j=1,2,3) as:

(1) $$\begin{array}{cc} {\dot{E}}_{ij}= & \frac{1}{V}\int_{V}{\dot{\varepsilon }}_{ij}\;dV\\ \Sigma_{ij}= & \frac{1}{V}\int_{V}\sigma_{ij}\;dV \end{array}$$

Here: *V* is the RVE volume; ${\dot{\varepsilon }}_{ij}$ and $\sigma_{ij}$ are the local (microscopic) strain rate and stress tensors, respectively.

Provided that elastic deformation inside the RVE can be disregarded, the macroscopic plastic dissipation can be written:

(2) $$\begin{array}{c} \dot{W}=\frac{1}{V}\int_{V}\dot{w}\;dV \end{array}$$

$\dot{w}=\sigma_{ij}{\dot{\varepsilon }}_{ij}$ being the local dissipation.

If an affine velocity field

(3) $$\begin{array}{c} v_{i}={\dot{E}}_{ij}x_{j}\;\text{on}\;\partial V \end{array}$$

$x_{i}$ denoting the position vector, has to be fulfilled on the outer boundary ∂V of the RVE [13,24,25], an upper bound on macroscopic yielding can be obtained as:

(4) $$\begin{array}{c} \Sigma_{ij}=\frac{\partial \dot{W}}{\partial {\dot{E}}_{ij}} \end{array}$$

where the the no-correlation postulate [26] has been exploited (see also [6]).

**Figure 1:**
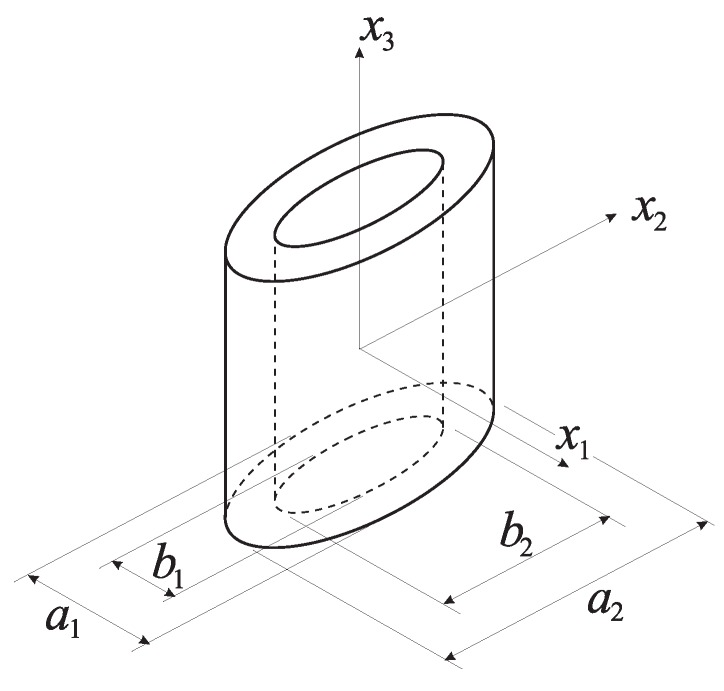
Geometry of the considered representative volume element.

Let us consider now a cylindrical RVE with elliptic cross-section, containing a coaxial and confocal elliptic cylindrical cavity, see Figure 1. This RVE does not fill the continuum without gaps, but approximately represents the actual microstructure of an array of hexagonal cylindrical void-containing RVEs with different void spacings along axes $x_{1}$ and $x_{2}$. The RVE microstructure is here described through the void volume fraction *f* and the aspect ratio *λ* of the void in its cross-section, which are respectively defined as (see Figure 1):

(5) $$f=\frac{V_{v}}{V}=\frac{\pi \frac{b_{1}b_{2}}{4}}{\pi \frac{a_{1}a_{2}}{4}}=\frac{b_{1}b_{2}}{a_{1}a_{2}}$$

(6) $$\lambda =\frac{b_{2}}{b_{1}}$$

where $V_{v}$ is the volume of the void. In Equations (5) and (6), $a_{\alpha }$ and $b_{\alpha }$ (α=1,2) respectively represent the axis lengths of the two (outer and inner) ellipses bounding the matrix phase.

To get an analytic expression for (4), the following coordinate mapping in the $x_{1}-x_{2}$ plane is introduced:

(7) $$\left\{\begin{array}{c} x_{1}=r\left(1-\frac{c}{r^{2}}\right)\sin\beta \\ x_{2}=r\left(1+\frac{c}{r^{2}}\right)\cos\beta \end{array}\right.$$

where confocality of the outer and inner ellipses is obtained with:

(8) $$c=\frac{a_{2}^{2}-a_{1}^{2}}{4}=\frac{b_{2}^{2}-b_{1}^{2}}{4}$$

The above mapping, which is similar to the three-dimensional axisymmetric one proposed in [27], can be adopted for ellipses featuring any aspect ratio, either λ>1 or λ<1. Obviously, the transformation (7) becomes singular as r→0; as shown in what follows, since plastic dissipation $\dot{w}$ has to be evaluated in the matrix only (r≠0), the aforementioned singularity does not represent an issue.

Assuming that the whole RVE plastically deforms at macroscopic yielding [6,11], solutions are sought under generalized plane strain conditions, *i.e.*, under a uniform strain rate in the $x_{3}$ direction. If the matrix material obeys $J_{2}$ plasticity theory, the velocity field at plastic collapse has to be divergence-free. To further satisfy the kinematic boundary condition (3) on ∂V, the cartesian components $v_{i}$ need to be, see [8,10]:

(9) $$\left\{\begin{array}{c} v_{1}=\frac{1}{2}\left[\frac{a_{1}a_{2}}{r^{2}-c}\left({\dot{E}}_{a}+{\dot{E}}_{33}\right)+{\dot{E}}_{b}-2\ell {\dot{E}}_{33}\right]x_{1}\\ v_{2}=\frac{1}{2}\left[\frac{a_{1}a_{2}}{r^{2}+c}\left({\dot{E}}_{a}+{\dot{E}}_{33}\right)-{\dot{E}}_{b}-2\frac{a_{1}}{a_{2}}\ell {\dot{E}}_{33}\right]x_{2}\\ v_{3}={\dot{E}}_{33}x_{3} \end{array}\right.$$

where:

(10) $$\begin{array}{c} \ell =\frac{a_{2}}{a_{2}+a_{1}} \end{array}$$

and:

(11) $$\begin{array}{cc} {\dot{E}}_{a}= & {\dot{E}}_{11}+{\dot{E}}_{22}\\ {\dot{E}}_{b}= & 2\ell \left(\frac{a_{1}}{a_{2}}{\dot{E}}_{11}-{\dot{E}}_{22}\right) \end{array}$$

The velocity field (9) leads to the following effective strain rate:

(12) $$\begin{array}{c} {\dot{\varepsilon }}_{eq}=\sqrt{\frac{2}{3}\left({\dot{\varepsilon }}_{11}^{2}+{\dot{\varepsilon }}_{22}^{2}+{\dot{\varepsilon }}_{33}^{2}\right)}=\sqrt{\frac{1}{3}{\dot{\Theta }}_{eq}} \end{array}$$

where:

(13) $$\begin{array}{cc} {\dot{\Theta }}_{eq}= & {\dot{E}}_{b}^{2}+4\left(\ell^{2}-\ell +1\right){\dot{E}}_{33}^{2}+2\left(1-2\ell \right){\dot{E}}_{b}{\dot{E}}_{33}\\ & +\frac{a_{1}a_{2}}{H}\left({\dot{E}}_{a}+{\dot{E}}_{33}\right)\left[2\left(r^{2}\cos2\beta -c\right)\left({\dot{E}}_{b}+\left(1-2\ell \right){\dot{E}}_{33}\right)+a_{1}a_{2}\left({\dot{E}}_{a}+{\dot{E}}_{33}\right)\right] \end{array}$$

and $H=r^{4}+c^{2}-2cr^{2}\cos2\beta$.

Now, by accounting for the null strength of the void and by neglecting hardening in the matrix (*i.e.*, by assuming the matrix to be rigid-perfectly plastic), the macroscopic plastic dissipation becomes [9,10]:

(14) $$\begin{array}{c} \dot{W}=\frac{\sigma_{0}}{V}\int_{\Omega }\sqrt{\frac{1}{3}{\dot{\Theta }}_{eq}}\;dV \end{array}$$

where: Ω is the matrix volume; $\sigma_{0}$ is the matrix yield strength under uniaxial loading.

Following the procedure proposed by Gurson for a circular cylindrical RVE [6], the plastic dissipation $\dot{W}$ is expanded in Taylor series about cos2β=0. This expansion, if arrested at the first order, under generalized plane strain conditions furnishes:

(15) $$\begin{array}{c} \dot{W}=\frac{2\sigma_{0}}{\sqrt{3}a_{1}a_{2}}\int_{r_{b}}^{r_{a}}\frac{r^{4}+c^{2}}{r^{3}}\sqrt{{\dot{\Theta }}_{eq}^{I}}\;dr \end{array}$$

where: *c* is given in Equation (8); $r_{b}=\frac{b_{1}+b_{2}}{2}$ and $r_{a}=\frac{a_{1}+a_{2}}{2}$; ${\dot{\Theta }}_{eq}^{I}$ is the value of ${\dot{\Theta }}_{eq}={\dot{\Theta }}_{eq}\left(\beta \right)$ computed at cos2β=0. The upper bound on the overall yield locus is given, in terms of macroscopic stress components conjugate to the strain rates ${\dot{E}}_{a}$, ${\dot{E}}_{b}$ and ${\dot{E}}_{33}$, by:

(16) $$\begin{array}{cc} \Sigma_{a} & =\frac{\partial \dot{W}}{\partial {\dot{E}}_{a}}=\frac{2\sigma_{0}}{\sqrt{3}a_{1}a_{2}}\int_{r_{b}}^{r_{a}}\frac{r^{4}+c^{2}}{r^{3}}\frac{{\dot{\Theta }}_{eqa}^{I}}{2\sqrt{{\dot{\Theta }}_{eq}^{I}}}\;dr\\ \Sigma_{b} & =\frac{\partial \dot{W}}{\partial {\dot{E}}_{b}}=\frac{2\sigma_{0}}{\sqrt{3}a_{1}a_{2}}\int_{r_{b}}^{r_{a}}\frac{r^{4}+c^{2}}{r^{3}}\frac{{\dot{\Theta }}_{eqb}^{I}}{2\sqrt{{\dot{\Theta }}_{eq}^{I}}}\;dr\\ \Sigma_{33} & =\frac{\partial \dot{W}}{\partial {\dot{E}}_{33}}=\frac{2\sigma_{0}}{\sqrt{3}a_{1}a_{2}}\int_{r_{b}}^{r_{a}}\frac{r^{4}+c^{2}}{r^{3}}\frac{{\dot{\Theta }}_{eq33}^{I}}{2\sqrt{{\dot{\Theta }}_{eq}^{I}}}\;dr \end{array}$$

where:

(17) $$\begin{array}{cc} {\dot{\Theta }}_{eqa}^{I}= & \frac{2a_{1}a_{2}}{r^{4}+c^{2}}\left\{a_{1}a_{2}\left({\dot{E}}_{a}+{\dot{E}}_{33}\right)-c\left({\dot{E}}_{b}+\left(1-2\ell \right){\dot{E}}_{33}\right)\right\}\\ {\dot{\Theta }}_{eqb}^{I}= & \frac{2}{r^{4}+c^{2}}\left\{\left(r^{4}+c^{2}\right)\left({\dot{E}}_{b}+\left(1-2\ell \right){\dot{E}}_{33}\right)-a_{1}a_{2}\left({\dot{E}}_{a}+{\dot{E}}_{33}\right)c\right\}\\ {\dot{\Theta }}_{eq33}^{I}= & \frac{2}{r^{4}+c^{2}}\{\left(r^{4}+c^{2}\right)\left(4\left(\ell^{2}-\ell +1\right){\dot{E}}_{33}+\left(1-2\ell \right){\dot{E}}_{b}\right)\\ & +a_{1}a_{2}\left[a_{1}a_{2}\left({\dot{E}}_{a}+{\dot{E}}_{33}\right)-c\left({\dot{E}}_{b}+\left(1-2\ell \right)\left({\dot{E}}_{a}+2{\dot{E}}_{33}\right)\right)\right]\} \end{array}$$

By finally exploiting the chain rule for differentiation, macroscopic stress components $\Sigma_{11}$ and $\Sigma_{22}$ at yielding turn out to be (see Equation (11)):

(18) $$\begin{array}{cc} \Sigma_{11} & =\frac{\partial \dot{W}}{\partial {\dot{E}}_{11}}=\frac{\partial \dot{W}}{\partial {\dot{E}}_{a}}\frac{\partial {\dot{E}}_{a}}{\partial {\dot{E}}_{11}}+\frac{\partial \dot{W}}{\partial {\dot{E}}_{b}}\frac{\partial {\dot{E}}_{b}}{\partial {\dot{E}}_{11}}=\Sigma_{a}+2\frac{a_{1}}{a_{2}}\ell \Sigma_{b}\\ \Sigma_{22} & =\frac{\partial \dot{W}}{\partial {\dot{E}}_{22}}=\frac{\partial \dot{W}}{\partial {\dot{E}}_{a}}\frac{\partial {\dot{E}}_{a}}{\partial {\dot{E}}_{22}}+\frac{\partial \dot{W}}{\partial {\dot{E}}_{b}}\frac{\partial {\dot{E}}_{b}}{\partial {\dot{E}}_{22}}=\Sigma_{a}-2\ell \Sigma_{b} \end{array}$$

This solution can be analytically expressed in terms of elliptic integrals of the first and second kind.

**Figure 2:**
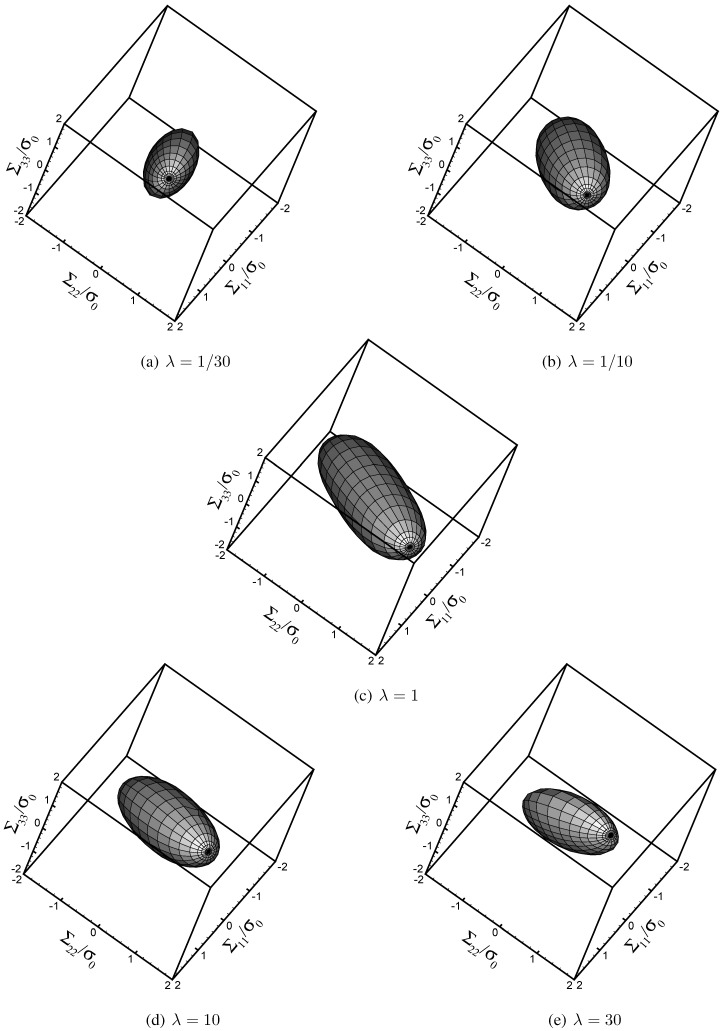
Overall yield condition in the $\Sigma_{11}-\Sigma_{22}-\Sigma_{33}$ space, at varying *λ* (f=0.05).

To get a picture of the effect of void shape on the homogenized yield locus in the $\Sigma_{11}-\Sigma_{22}-\Sigma_{33}$ space, outcomes are shown in Figure 2 at varying void aspect ratio ($\lambda =\frac{1}{30},\frac{1}{10},1,10,30$) and assigned porosity (f=0.05). It appears that aspect ratios different from the customarily adopted value λ=1 act by reducing the effective strength of the void-containing material, *i.e.*, by reducing the size of the overall yield locus. As already highlighted in [10], this strength reduction mainly occurs along the minor principal axis of the elliptic cross-section of the void.

Concerning microstructure evolution at yielding, exploitation of matrix incompressibility leads to the following evolution law for the porosity *f*:

(19) $$\begin{array}{c} \dot{f}=\left(1-f\right)\frac{{\dot{V}}_{v}}{V}=\left(1-f\right)\left({\dot{E}}_{a}+{\dot{E}}_{33}\right) \end{array}$$

The void aspect ratio *λ* instead evolves according to:

(20) $$\begin{array}{cc} \dot{\lambda }={\left(\frac{b_{2}}{b_{1}}\right)}^{\cdot }= & \frac{{\dot{b}}_{2}-\lambda {\dot{b}}_{1}}{b_{1}}\\ = & \frac{\lambda }{f}\frac{1-\lambda }{1+\lambda }\left({\dot{E}}_{a}+{\dot{E}}_{33}\right)-\lambda {\dot{E}}_{b}-\lambda \frac{1-\kappa_{v}}{1+\kappa_{v}}{\dot{E}}_{33} \end{array}$$

where: ${\dot{b}}_{1}=v_{1}\left(x_{1}=b_{1},x_{2}=0\right)$, ${\dot{b}}_{2}=v_{2}\left(x_{1}=0,x_{2}=b_{2}\right)$, see Equation (9); and:

(21) $$\begin{array}{c} \kappa_{v}=\left(\frac{f}{2}\frac{\lambda^{2}-1}{\lambda }\right)+\sqrt{1+{\left(\frac{f}{2}\frac{\lambda^{2}-1}{\lambda }\right)}^{2}} \end{array}$$

Handling both the evolution laws (19) and (20) allows to describe microstructure evolution induced by deviatoric states of stress, as reported in the forthcoming Section 3. Under such loading conditions, which generally lead to ${\dot{E}}_{a}+{\dot{E}}_{33}\approx 0$, the Gurson’s model proves deficient since it furnishes $\dot{f}\approx 0$ and can not describe the softening, and the subsequent propagation of macroscopic cracks, primarily induced by void distortion.

## 3. Microstructure Evolution and Overall behavior of Orthotropic Porous-Ductile Media

The accuracy of the yield locus for anisotropic mildly-voided porous-plastic solids is now assessed through comparison with outcomes of finite element simulations at unit-cell level. Results obtained with the Gurson’s model, which always assumes λ=1 and disregards void shape evolution caused by deformation, are also used as a term of comparison.

Throughout this Section we assume that plane strain conditions occurs; such kinematic conditions are of primary importance for ductile fracture, since they are always attained ahead of the central portion of any growing crack front (axis $x_{3}$ being assumed locally aligned with the crack front).

An elastic-perfectly plastic matrix material is considered, featuring $E/\sigma_{0}=400$ and ν=0.3 (E being the Young’s modulus and *ν* the Poisson’s ratio). Since we are here interested in the response of the porous-plastic solid at finite strains (characterized by logarithmic strains on the order of 1), the elastic properties of the matrix (which mainly affect the response of the solid up to logarithmic strains on the order of $10^{-2}$) play a minor role. Therefore, at the constitutive level we do not account for the effect of microstructure evolution on the homogenized elastic properties of the void-containing material, which are obtained in closed-form with the self-consistent scheme as (see [28], Chapter 5):

(22) $$\begin{array}{cc} E_{1}=E_{2} & =(1-3f_{0})E\\ E_{3} & =(1-f_{0})E\\ \nu_{12} & =\nu -\left(3\nu -1\right)f_{0} \end{array}$$

where: $f_{0}$ is the initial material porosity, in the undeformed state; $E_{1}$, $E_{2}$ and $E_{3}$ are the effective Young’s moduli (in the $x_{1}$, $x_{2}$ and $x_{3}$ directions) of the porous material; $\nu_{12}$ is the effective Poisson’s ratio linking deformations in the $x_{1}-x_{2}$ plane. These estimates are expected to be accurate for small values of the porosity $f_{0}$. As already highlighted in what precedes, the offered constitutive description for porous-plastic solids, which assumes that plastic dissipation is spread all over the matrix, is valid only up to the critical threshold f≈0.2, which meets the aforementioned dilute void content requirement.

Equation (22) rule out damage-like dissipative mechanisms at the macroscale, which should be linked to the continuously reducing effective Young’s moduli due to void growth. Microstructure evolution is therefore assumed to be induced by plastic deformations only.

As far as computational aspects are concerned, a second-order Runge-Kutta stepping procedure has been adopted to time-integrate the constitutive law. Since the yield locus (16)-(18) is furnished in a parametric fashion, a specifically devised algorithm needs to be developed; for the sake of brevity, and since these details are beyond the scope of the paper, readers are referred to [9] for details.

Considering strain-driven loading processes, the nonlinear response of the void-containing solid is investigated under assigned strain histories of the following type:

(23) $$\varrho_{2}-1=k_{\varepsilon }\left(\varrho_{1}-1\right)$$

where: $\varrho_{1}$ and $\varrho_{2}$ are, respectively, the stretch ratios [29] along axes $x_{1}$ and $x_{2}$; $k_{\varepsilon }$ is a parameter that can be finely tuned to affect the simultaneous changes of void size and shape, and that is kept constant during each analysis. All these strain paths depart from the undeformed state $\varrho_{1}=\varrho_{2}=1$.

**Figure 3:**
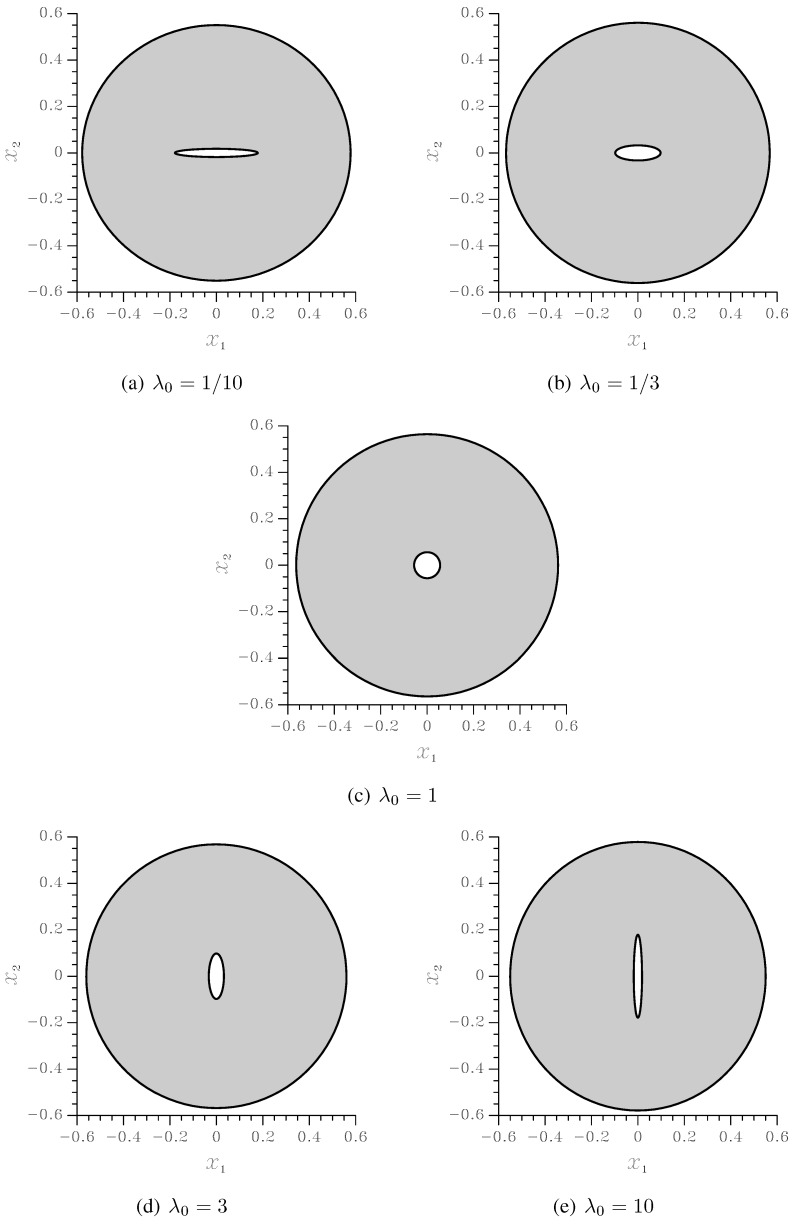
Undeformed RVE cross-sections at $f_{0}=0.01$ and varying void aspect ratio $\lambda_{0}$ (measures in mm). As a reference, in this figure the volume *V* (see Equations 1) and the out-of-plane thickness *L* of the RVE has been respectively assumed V=1 mm${}^{3}$ and L=1 mm.

To delay any coalescence-dominated event as much as possible, the initial void volume fraction has been set to $f_{0}=0.01$. The initial void aspect ratio has been instead assumed $\lambda_{0}=\frac{1}{10},\frac{1}{3},1,3,10$, in order to get much insights into the effect of void shape on the softening regime, which physically precedes failure by void coalescence. The undeformed RVE cross-sections relevant to the just mentioned values of $f_{0}$ and $\lambda_{0}$ are depicted in Figure 3; since the two elliptical surfaces bounding the matrix are forced to be confocal, the distribution of the voids in the $x_{1}-x_{2}$ plane turns out to be marginally anisotropic. To investigate the effect of highly-anisotropic void distributions, this confocality requirement therefore needs to be avoided.

Finite element unit-cell simulations have been run by space discretizing the RVE cross-section (actually only one quarter by exploiting the symmetries), and by applying on the outer surface ∂V an affine displacement field obtained by time integration of the boundary condition (3). In this work constitutive modeling and unit-cell calculations are compared for the assigned RVE geometries depicted in Figure 3; this has been devised to avoid accounting for adjusting parameters (like the Tvergaard’s ones [2,30]), usually introduced to prove the transferability of micromechanical studies when unit-cell geometries perfectly filling the continuum are adopted, (e.g., see [31]). In the simulations, the characteristic size $h_{e}$ of the elements has been set by checking that a reduction of $h_{e}$ did not lead to any variations in the simulated material response throughout the whole analysis.

To assess the accuracy of the proposed yield locus, we investigate the material response under $k_{\varepsilon }=0.0$ and $k_{\varepsilon }=-0.5$: the former strain path leads to a continuously increasing *f*, and therefore allows to check the model accuracy when the aforementioned critical threshold f≈0.2 for the void volume fraction is approached; the latter strain path instead causes void increase followed by a quite sharp decrease linked to a shaping of the void as a needle-like cavity, and therefore allows to check the model accuracy when void distortion effects become dominant.

**Figure 4:**
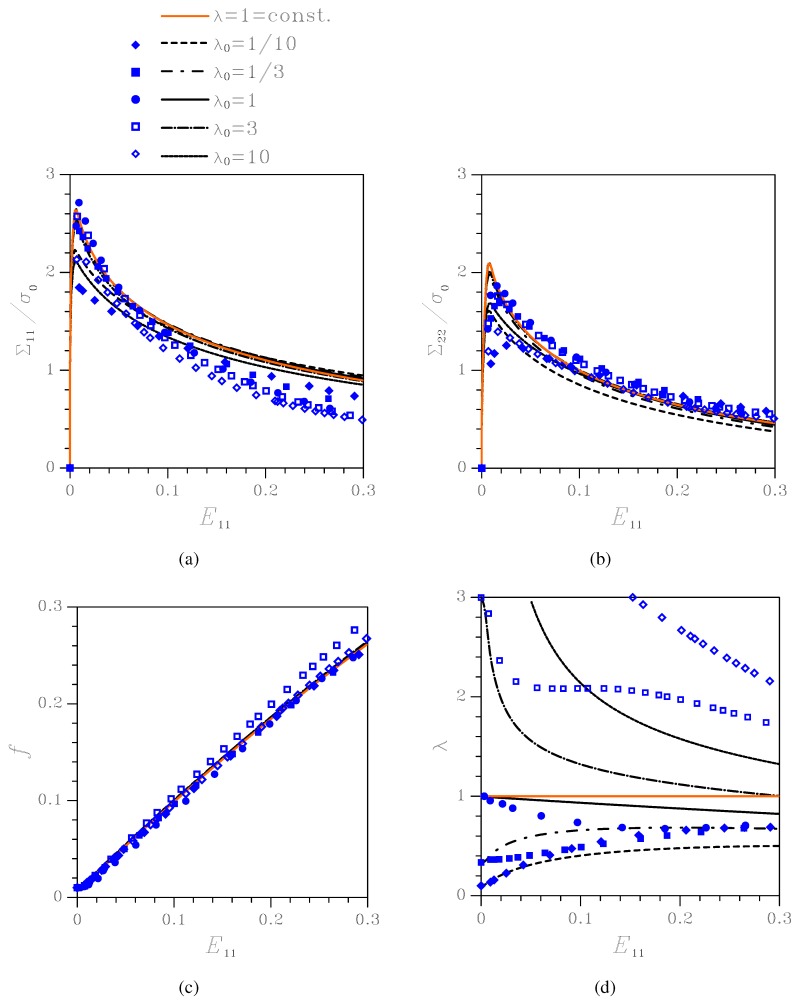
Effect of the initial aspect ratio $\lambda_{0}$ on the nonlinear response of the orthotropic porous-plastic solid at $k_{\varepsilon }=0.0$. Comparison among the proposed constitutive model (black lines), the Gurson’s model (orange line), and unit-cell simulations (blue symbols) in terms of: (a) Cauchy stress $\Sigma_{11}$ *vs* logarithmic strain $E_{11}$; (b) Cauchy stress $\Sigma_{22}$ *vs* logarithmic strain $E_{11}$; (c) void volume fraction *f* *vs* $E_{11}$; (d) void aspect ratio *λ* *vs* $E_{11}$.

**Figure 5:**
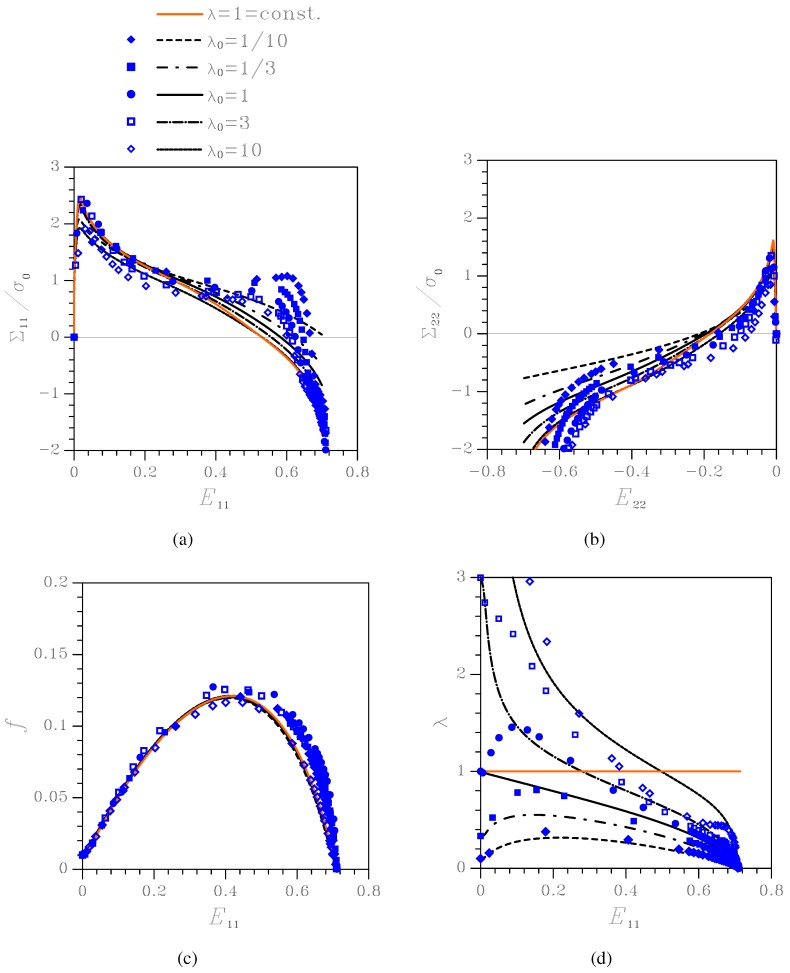
Effect of the initial aspect ratio $\lambda_{0}$ on the nonlinear response of the orthotropic porous-plastic solid at $k_{\varepsilon }=-0.5$. Comparison among the proposed constitutive model (black lines), the Gurson’s model (orange line), and unit-cell simulations (blue symbols) in terms of: (a) Cauchy stress $\Sigma_{11}$ *vs* logarithmic strain $E_{11}$; (b) Cauchy stress $\Sigma_{22}$ *vs* logarithmic strain $E_{22}$; (c) void volume fraction *f* *vs* $E_{11}$; (d) void aspect ratio *λ* *vs* $E_{11}$.

Figure 4 shows material response and microstructure evolution under $k_{\varepsilon }=0.0$, namely under uniaxial deformation with constrained lateral strains. Results are presented in terms of: Cauchy stress components $\Sigma_{11}$ and $\Sigma_{22}$ *vs* logarithmic strain $E_{11}$ (since here $E_{22}=0$ throughout the whole deformation process, $\Sigma_{22}$ is plotted *vs* $E_{11}$); evolution of the state variables *f* and *λ*. The results obtained by time-integration of the proposed constitutive model are compared to those furnished by the transversely isotropic Gurson’s model (termed λ=1=const. in the plots), which assumes that the void aspect ratio *λ* does not evolve, and to those relevant to unit-cell simulations. To understand the major discrepancies between the constitutive description and unit-cell simulations, specially when localized deformation modes, subsequently leading to coalescence, set in, results are plotted as long as f≤0.25.

As far as the stress-strain relationships are concerned, $\lambda_{0}$ plays a role by mainly affecting the peak stress values, at the inception of the softening stage; this aspect is further discussed next. Void growth is instead only marginally affected by $\lambda_{0}$. The aspect ratio histories show that, in all the cases featuring $\lambda_{0}\neq 1$ the voids mainly evolve toward a circular cross-section.

Figure 5 shows material response and microstructure evolution under $k_{\varepsilon }=-0.5$, namely under simultaneous positive stretching along axis $x_{1}$ ($E_{11}>0$) and negative stretching along axis $x_{2}$ ($E_{22}<0$). Outcomes testify that *f* starts increasing as soon as the elastic limit is attained but, at $E_{11}≅0.44$ it decreases due to the imposed deformation path, and eventually becomes null at $E_{11}≅0.71$; this evolution of *f*, like in the $k_{\varepsilon }=0.0$ case, is almost unaffected by $\lambda_{0}$. Because of the evolution of *f*, the $\Sigma_{11}$ *vs* $E_{11}$ and $\Sigma_{22}$ *vs* $E_{22}$ responses are eventually characterized by a hardening stage in compression. While the compressive $\Sigma_{22}$ is clear to understand, a compressive $\Sigma_{11}$ is less obvious and turns out to be linked to the volume-preserving J${}_{2}$ constitutive law of the matrix material. To avoid the overall compression in the $x_{1}$ direction, the strain $E_{11}$ should be increased more rapidly when the void volume fraction is approaching f=0, thereby not fulfilling the requirement of constant $k_{\varepsilon }$ during the whole analysis. Figure 5(d) also depicts that *f* vanishes at $E_{11}≅0.71$ because *λ* tends to zero, that is because the void tends to get shaped like a needle; obviously, this feature cannot be described by the Gurson’s model, which assumes λ=1 at any deformation level.

**Figure 6:**
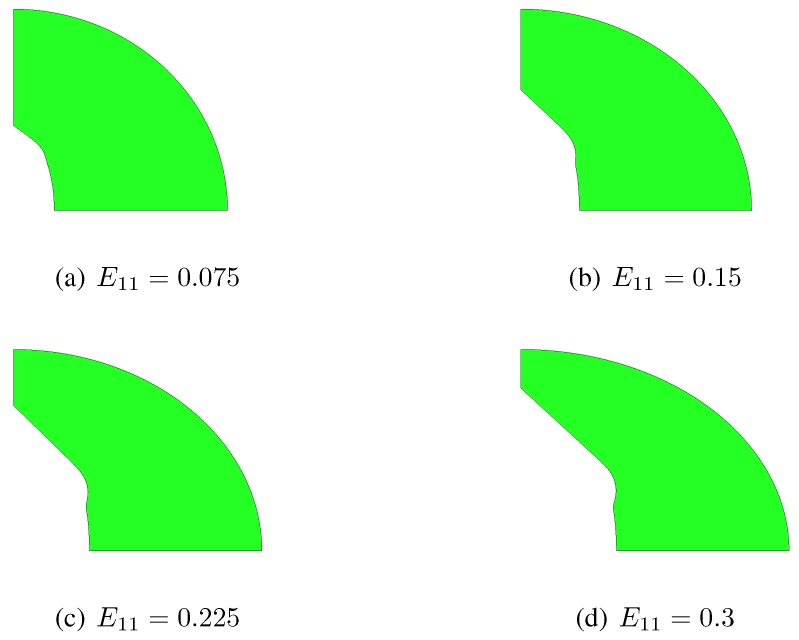
$f_{0}=0.01$, $\lambda_{0}=3$. Microstructure evolution under $k_{\varepsilon }=0.0$, as obtained with the unit-cell simulation.

**Figure 7:**
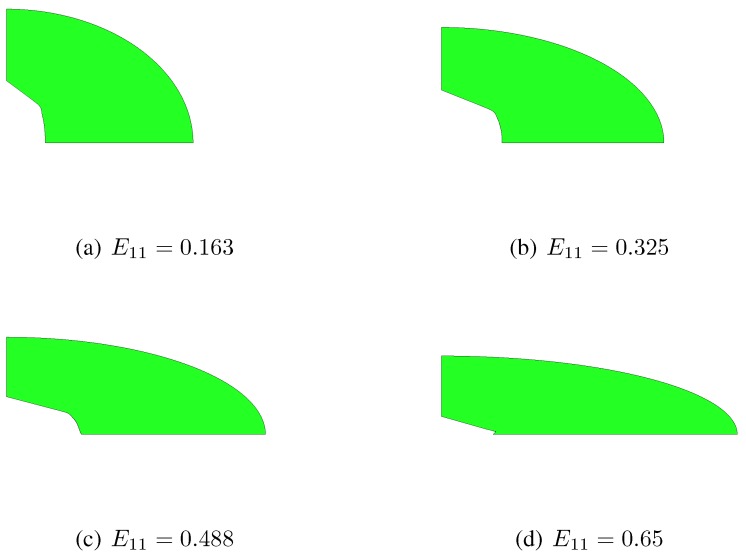
$f_{0}=0.01$, $\lambda_{0}=3$. Microstructure evolution under $k_{\varepsilon }=-0.5$, as obtained with the unit-cell simulation.

When the proposed constitutive description is compared to unit-cell simulations, the main difference shows up in the evolution of the void aspect ratio, which also causes a slight time discrepancy in the evolution of the void volume fraction. In the finite element simulations the void looses its elliptical shape while growing; besides the deformation modes considered in Section 2, additional localized ones are therefore incepted, independently of the strain path. This is testified by Figure 6 and Figure 7, which respectively show snapshots of the RVE evolution under $k_{\varepsilon }=0.0$ and $k_{\varepsilon }=-0.5$, in the case $\lambda_{0}=3$. These figures further show that, while the constitutive model assumes the two ellipses bounding the matrix to be always confocal, they actually evolve losing confocality; this phenomenon turns out to be a further cause of discrepancy between constitutive model and unit-cell simulations. As already discussed in [10], the discrepancy grows for aspect ratios far different from λ=1, since the actual plastic collapse of the RVE becomes more affected by localized yielding modes.

**Figure 8:**
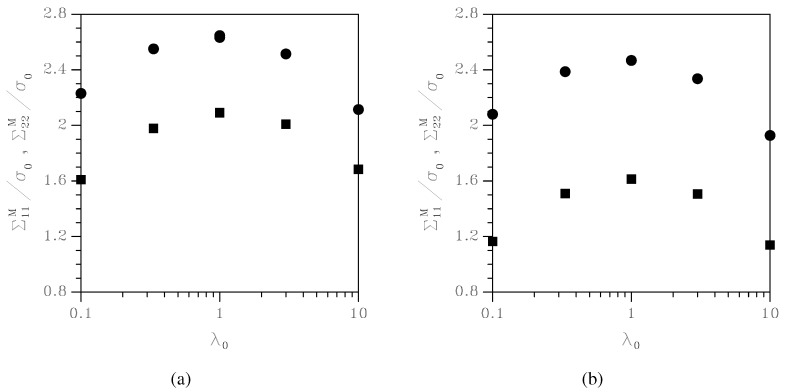
Effect of the initial aspect ratio $\lambda_{0}$ on the peak values of Chauchy stresses $\Sigma_{11}$ (circles) and $\Sigma_{22}$ (squares), under (a) $k_{\varepsilon }=0.0$, and (b) $k_{\varepsilon }=-0.5$.

We highlighted above that non-circular void cross-sections cause a reduction of the strength of the porous-plastic solid; the proposed model allows to evaluate this effect. Figure 8 collects plots of the peak values of the Cauchy stresses $\Sigma_{11}$ and $\Sigma_{22}$ as a function of $\lambda_{0}$: under both $k_{\varepsilon }=0.0$ and $k_{\varepsilon }=-0.5$, it can be seen that strongly elliptical voids can affect the overall strength of the porous-plastic solid by more than 30%. In orthotropic porous-plastic solids, this strength reduction may therefore easily trigger strain localization, and subsequent ductile fracture nucleation.

## 4. Concluding Remarks

In this paper we have proposed a micromechanical analysis of a two-phase composite, constituted by a ductile matrix surrounding a void (treated as an inclusion with null elastic and strength properties). This study is aimed at assessing how the evolution in shape and size of the void can affect the overall response of orthotropic porous-plastic solids. It is in fact known that void growth to coalescence is the main micromechanical process leading to ductile fracture, but the effects of void distortion in the process zone ahead of the tip of growing cracks are usually disregarded or approximately accounted for by existing constitutive models.

A nonlinear homogenization procedure based on the kinematic approach to limit analysis has been followed, and an upper bound on the overall yield locus for the void-containing material has been obtained. By exploiting all the features of the collapse mechanism, plasticity-driven evolution laws for the void volume fraction (*i.e.*, void size) and the void aspect ratio (*i.e.*, void shape) have been obtained too.

To assess the capability of the offered solution and to describe the strength reduction due to void growth, the constitutive response and the microstructure evolution have been investigated under assigned strain histories. It has been shown that, under predominantly deviatoric states of stress, circular-cylindrical voids may evolve into zero-volume needles; this outcome can not be described at the constitutive level by the usually adopted Gurson’s model [6], which instead assumes that the shape of the void is never affected by loading. Moreover, anisotropic microstructures have shown to cause a reduction of the overall strength of the void-containing material; this means that the Gurson’s model can overestimate the actual stress-carrying capacity of porous-plastic solids.

Through comparison with finite element unit-cell simulations, we have also assessed the accuracy of the proposed constitutive law in capturing the distortion of the void at finite strains. It has been shown that, even though localized deformation modes (which can not be described by the model) show up in the matrix, the strength and the softening regime are both pretty well captured.

It has been remarked that one of the causes of the discrepancy between constitutive description and unit-cell simulations is the assumption of confocal ellipses bounding the matrix phase throughout the whole deformation process. In future developments, the offered solution needs therefore to be enhanced by allowing for non-confocal ellipses, each one able to evolve according to the applied loading condition and to the matrix behavior.
